# Guiding acute repair decisions of MCL injuries with a novel MRI classification system: a retrospective study of 226 cases with MRI and intraoperative findings

**DOI:** 10.3389/fsurg.2026.1808791

**Published:** 2026-05-14

**Authors:** Yu Mei, Shuangshuang Li, Zegang Wang, Xing Xie, Yu Yin, Dai Li, Shaofeng Yang

**Affiliations:** 1 Department of Sports Medicine, Peking University Third Hospital, Institute of Sports Medicine of Peking University, Beijing, China; 2 Beijing Key Laboratory of Research and Translation for Drugs and Medical Devices in Precision Diagnosis and Treatment of Sports Injuries, Beijing, China; 3 Engineering Research Center of Sports Trauma Treatment Technology and Devices, Ministry of Education, Beijing, China; 4 School of Kinesiology and Health, Capital University of Physical Education and Sports, Beijing, China

**Keywords:** diagnosis, medial collateral ligament (MCL) injury, MRI classification, repair, tear location

## Abstract

**Objective:**

This study aimed to investigate the relationship between magnetic resonance imaging (MRI) findings and intraoperative findings of Grade III medial collateral ligament (MCL) injuries to validating the diagnostic accuracy of MRI in localizing the tear and exploring the distribution of tear locations in these injuries.

**Methods:**

We reviewed 226 patients with MCL repair surgery and introduced a novel MRI-based classification system. MCL tears were classified into types 1a, 1b, 2a, 2b, 3, 4a, and 4b based on their location relative to the seven attachments. We then recorded tear locations and calculated the incidence of each type. The Kappa statistic was used to assess both the agreement between MRI and intraoperative classifications and inter-observer reliability. The sensitivity, specificity and accuracy (95% CIs) of MRI for localizing tears were calculated against intraoperative findings.

**Results:**

There was good inter-observer (*k* = 0.63, *P* < 0.001) and substantial intraoperative (*k* = 0.92, *P* < 0.001) agreement for the MRI classifications. MRI demonstrated high diagnostic performance across all locations. Sensitivity ranged from 81.8% (95% CI: 47.8%–96.8%) for location S2 to 100% for locations P1 (95% CI: 97.0%–100.0%) and P2 (95% CI: 75.9%–100.0%). Specificity was consistently high, ranging from 90.8% (95% CI: 80.3%–96.2%) for D1 to 100.0% (95% CI: 97.8%–100%) for S2. Accuracy ranged from 95.6% (95% CI: 92.1%–97.6%) for D1 to 99.6% (95% CI: 97.5%–99.9%) for P1 and P2. Type 1b tears were the most common (63.3%), followed by types 4b and 3. The posterior oblique ligament (POL) tears were present in 77.0% of the cohort.

**Conclusion:**

The MRI classification system provides high diagnostic accuracy in locating MCL tears, particularly POL tears, for which the adductor tubercle serves as a reliable reference. Thus, this system confirms that the presence of a POL tear is a key factor guiding acute MCL repair.

## 1 Introduction

Medial collateral ligament (MCL) injury is one of the most common ligament injuries of the knee (1–7). In a 10-year observational study of athletes, it was found that medial collateral ligament tears accounted for 7.9% of all knee injuries (5). The severity of MCL injuries varies, leading to challenges in diagnostic and treatment decisions. Misdiagnosis or delayed treatment may result in persistent instability, post-traumatic osteoarthritis, pain, or impairment of function (8, 9). Therefore, accurately assessing the extent and location of the MCL injury is critical to determining the most appropriate treatment plan.

The current diagnosis of MCL injury severity is primarily based on subjective clinical assessment, with the knee valgus stress test being the most appropriate tool (5, 10–12). Grade III MCL injuries with valgus laxity at both 0° and 30° of knee flexion are generally considered an indication for surgery (9, 13–16), whereas grade I and II injuries commonly heal with conservative treatment (17, 18). Early intervention with repair of the MCL, typically within 7–10 days of the injury (19, 20), may reduce surgical morbidity and avoid the need for graft harvesting (17). However, the diagnosis of a Grade III MCL injury, especially in the acute phase, is challenging and may be confounded by factors like joint swelling, pain, the doctor's experience and other knee conditions like meniscal tears or anterior cruciate ligament injuries (21). Although examination under anesthesia provides an accurate assessment of MCL injury (18, 22), its use is limited to cases where the need for surgery has already been clearly established, such as in cruciate ligament reconstruction. In this setting, the MCL can be evaluated intraoperatively to guide the final surgical plan.

Magnetic resonance imaging (MRI) provides detailed images of the knee joint and is increasingly used to diagnose MCL injuries (11, 23–25). Previous studies have primarily focused on detecting edema and tears surrounding the ligament (19), but the exact location of the MCL tear has not always been clearly described (11). MRI assessment of medial collateral ligament injuries is increasingly focused on the involvement of the attachments, specifically evaluating tears at the femoral and tibial attachments (2, 3, 25). Studies suggested that MCL tears at the distal tibia attachment may require operation (3, 23). Von Rehlingen-Prinz et al. (26, 27) recently proposed and validated an MRI-based classification system for superficial medial collateral ligament (sMCL) tears, categorizing them into six types based on anatomical location. They reported the distribution frequency of sMCL tear types and highlighted the potential influence of tear location on treatment strategy. However, the relationship between specific tear locations and the indication for surgery was not clarified, and the deep medial collateral ligament (dMCL) and posterior oblique ligament (POL), both integral components of the medial collateral ligament, were not discussed in terms of their tear locations and patterns (28).

This study aimed to investigate the relationship between MRI findings and intraoperative findings of Grade III MCL injuries to validating the diagnostic accuracy of MRI in localizing the tear and exploring the distribution of tear locations in these injuries. It can facilitate MRI diagnosis of Grade III injuries and thereby guide acute MCL repair.

## 2 Materials and methods

This research protocol was reviewed and approved by the institutional ethics committee. A waiver of informed consent was granted, as the study was retrospective.

**Figure 1:**
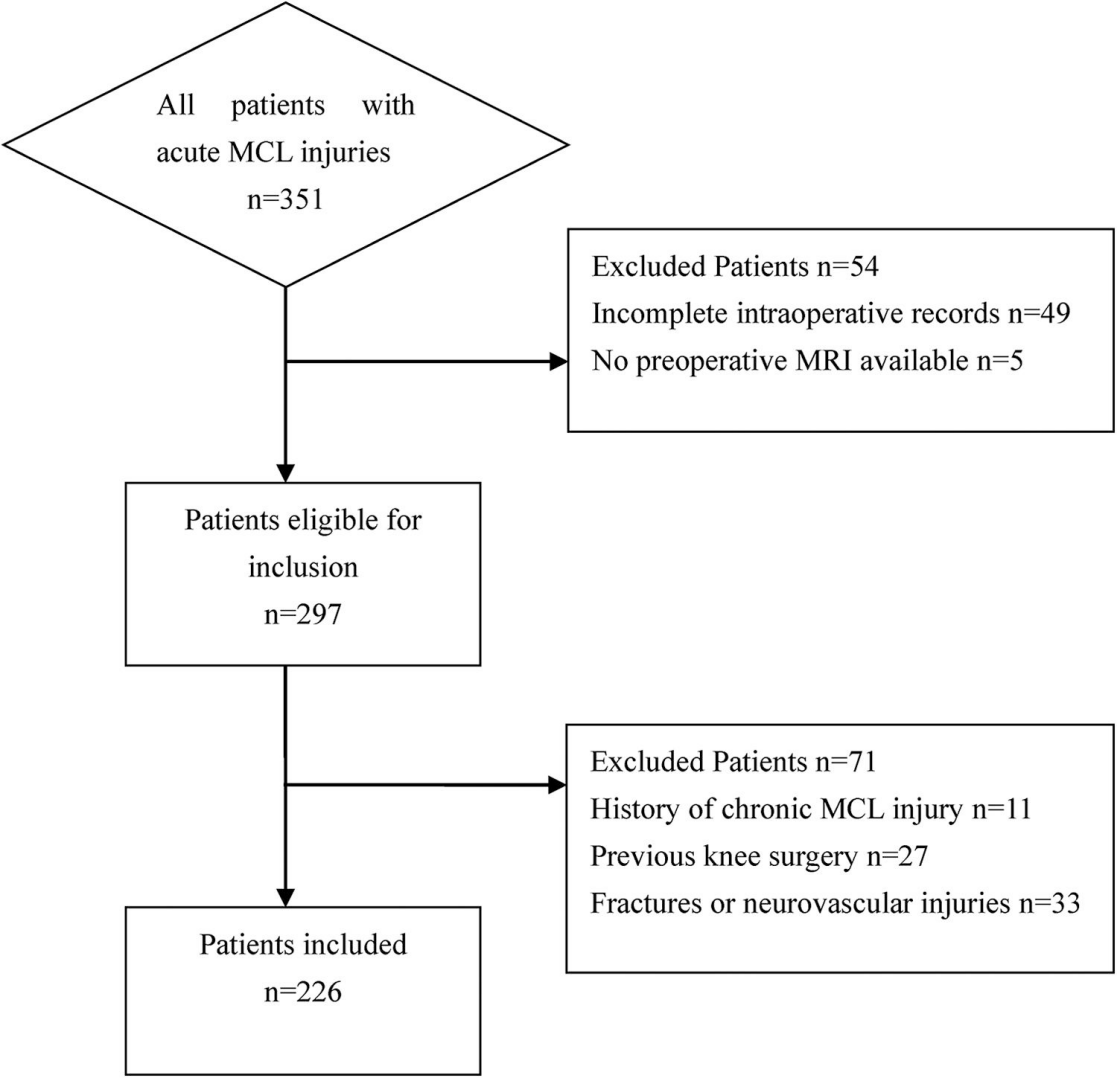
Flowchart of the studied population.

This retrospective study reviewed patients who underwent acute repair surgery for the MCL between January 2020 and December 2024. Inclusion criteria were: (1) Acute-phase MCL injury with a clear traumatic mechanism. (2) Complete preoperative magnetic resonance imaging (MRI) available. (3) Complete operative record available post-surgery. Exclusion criteria were: (1) History of chronic MCL injury. (2) History of knee fracture. (3) Previous knee surgery. (4) Concurrent neurovascular injury. Initially, 351 patients were identified. After applying the inclusion and exclusion criteria, 125 patients were excluded, leaving 226 patients for final analysis (Figure 1).

### 2.1 MRI imaging

MRI was performed using a 1.5T MRI scanner (GE Signa Explorer; *n* = 129) or a 3T scanner (Siemens MAGNETOM Lumina; *n* = 97). Although the scanners differed in magnetic field strength and manufacturer, both scanners operated under our institution's unified standardized knee MRI protocol. A prospective controlled study has confirmed that there is no significant difference in diagnostic value between 1.5T and 3.0T MRI from different manufacturers for characterizing knee ligament tears (29). Coronal images were acquired for both scanners and evaluated in conjunction with axial sequences. The imaging protocol specifically focused on two sequences: T1-weighted imaging (T1WI) and proton density-weighted imaging with fat saturation (PD-FS). Acquisition parameters for the 1.5T scanner were as follows: repetition time (TR) 2,342 ms, echo time (TE) 41.6 ms, field of view (FOV) 18 × 18 cm, matrix 320 × 256, and slice thickness 4.0 mm with a 0.4 mm gap. For the 3T scanner, parameters were: TR 2,830 ms, TE 44.0 ms, FOV 16 × 16 cm, matrix 384 × 269, and slice thickness 3.5 mm with a 0.5 mm gap.

### 2.2 MRI analysis

**Figure 2:**
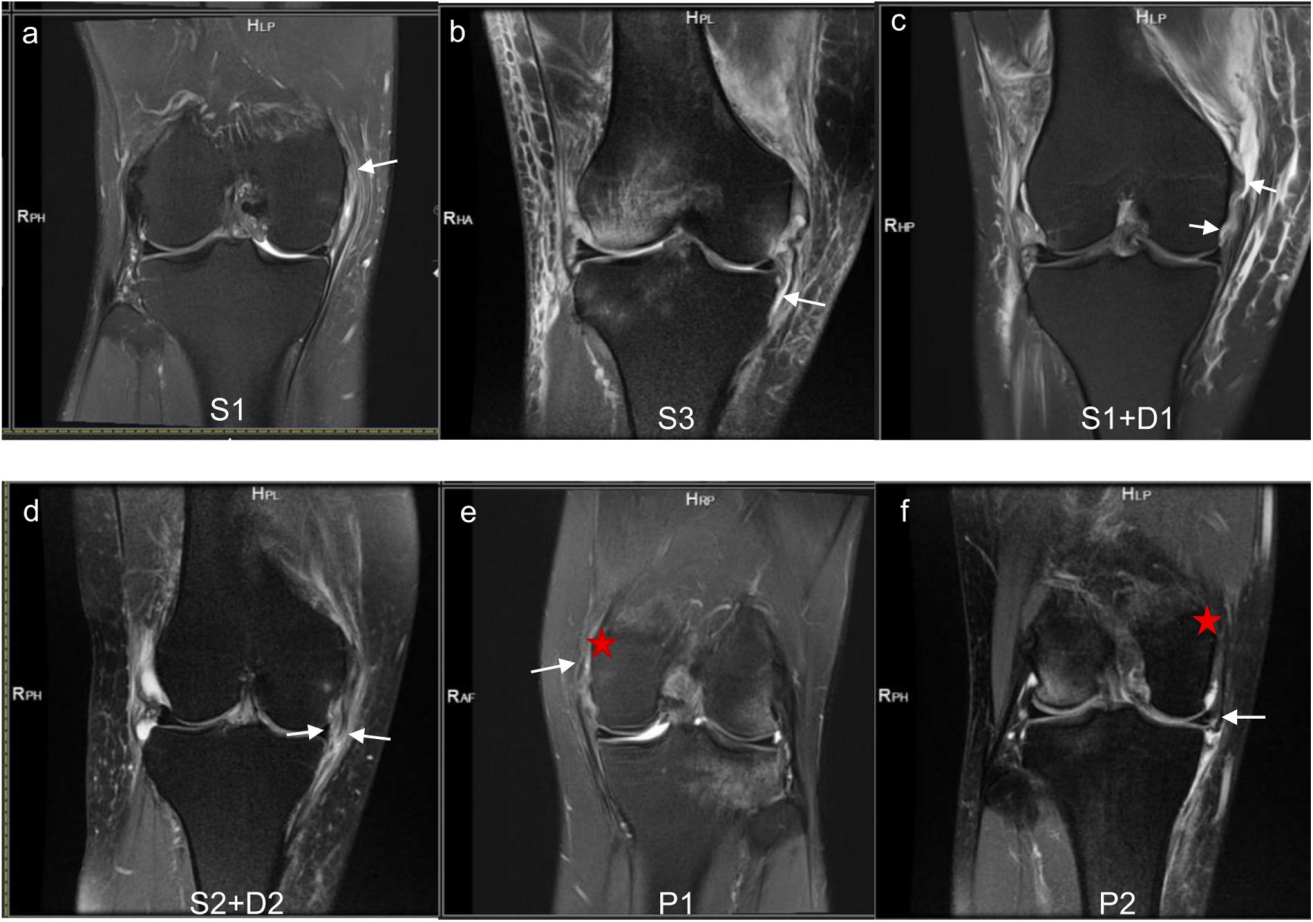
Coronal fat-saturated proton density-weighted (PDFS) magnetic resonance images demonstrate the tear areas of the medial collateral ligament (MCL). Image **(a)** shows a tear at the femoral side of the superficial medial collateral ligament (sMCL) (solid arrow). Image **(b)** reveals a tear at the distal tibial side of the sMCL (solid arrow). Image **(c)** demonstrates a combined tear at the femoral sides of both the sMCL and deep medial collateral ligament (dMCL) (solid arrow). Image **(d)** shows a concurrent tear at the proximal tibial sides of both the sMCL and dMCL (solid arrow). Image **(e)** shows a tear at the femoral side of the POL (solid arrow). **(f)** displays a tear at the tibial side of the POL (solid arrow). The adductor tubercle is indicated by an asterisk.

**Figure 3:**
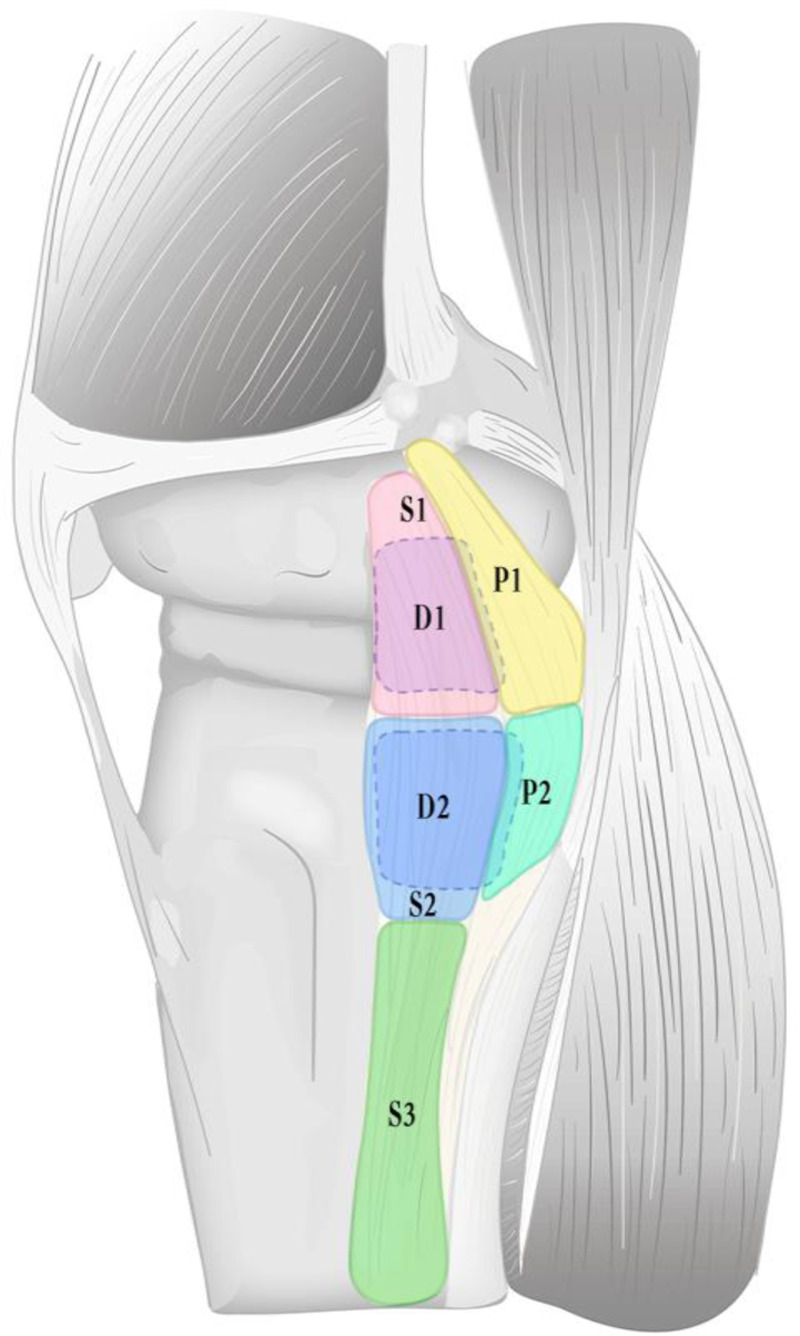
Division of the medial collateral ligament (MCL) into seven anatomical locations (This is an original schematic diagram created by the authors).

**Figure 4:**
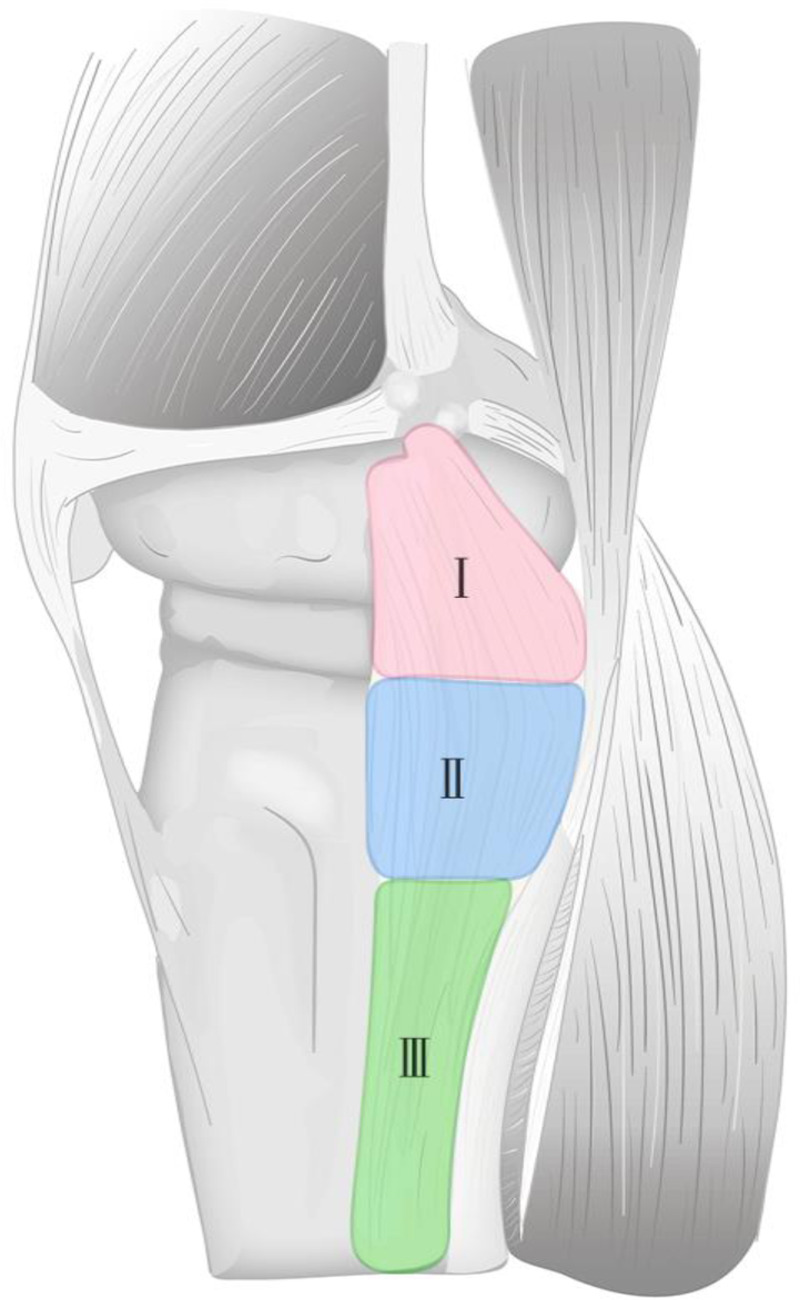
The proposed area classification for the medial collateral ligament (MCL) tears (This is an original schematic diagram created by the authors).

A tear was defined as a complete loss of fiber continuity, presenting as a localized grade III signal on MRI (19, 30), and its location was recorded (Figure 2). The sMCL and dMCL lie anterior to the adductor tubercle, while the posterior aspect is the POL (Figure 2). The MCL was divided into seven anatomical locations based on its attachments to the femur and tibia (Table 1 and Figure 3). These seven attachments are distributed across three areas: the femoral side, proximal tibial side, and distal tibial side. Accordingly, we classified them as types 1, 2, and 3, respectively, with tears affecting multiple regions categorized as type 4. Tears involving the sMCL and/or dMCL were classified as subtype a, while those involving the POL were classified as subtype b (Table 2 and Figure 4).

**Table 1:** MCL tear locations based on seven attachments.

MCL	Femoral side	Tibial side	
sMCL	S1	S2 (proximal tibial side)	S3 (distal tibial side)
dMCL	D1	D2	
POL	P1	P2 (distal tibial side or semimembranosus side)	

**Table 2:** Classification and definition of MCL tears on MRI.

Classification Type	Type Definition	Characteristic Injuries
Type 1	Femoral-side MCL tear	–
1a	Femoral-side tears of the sMCL and/or dMCL	S1, D1, S1 + D1
1b	Femoral-side tears involving the POL and the sMCL and/or dMCL	1a + P1
Type 2	Proximal tibial-side MCL tear	–
2a	Proximal tibial-side tears of the sMCL and/or dMCL	S2, D2, S2 + D2
2b	Proximal-side tibial tears involving the POL and the sMCL and/or dMCL	2b + P2
Type 3	Distal tibial-side MCL tear	S3
Type 4	Multi-region MCL tears	–
4a	Multi-region tears of the sMCL and/or dMCL	1a + 2a, 1a + 3, 2a + 3, 1a + 2a + 3
4b	Multi-region tears involving the POL and the sMCL and/or dMCL	4a + P1/P2

To evaluate inter-rater reliability, a random sample of 55 cases was selected from the total cohort of 226. An orthopaedic knee surgeon and a musculoskeletal radiologist independently interpreted these 55 MRI scans. All MRI interpretations were performed blinded to clinical gradings and intraoperative findings. For the remaining 171 cases, and for any discrepant readings identified during the reliability analysis, the two observers reviewed the images together and reached a consensus interpretation through discussion. This consensus interpretation for all 226 cases was then used for the subsequent diagnostic accuracy analysis against the intraoperative findings.

### 2.3 Intraoperative findings

The intraoperative records of all included patients were reviewed. The MCL tears were described in detail by the operating surgeons based on the actual intraoperative findings. Given the retrospective nature of the study, the surgeons were unaware of the classification system used in this analysis. Instead, they provided descriptive accounts of the tear location and extent as part of their routine clinical documentation, faithfully reflecting the intraoperative findings. These records were subsequently reviewed by a single researcher who was blinded to the preoperative MRI findings, and the tear locations were recorded and classified according to the descriptions provided. These intraoperative findings served as the reference standard for MRI diagnosis.

### 2.4 Statistical analysis

SPSS 26.0 statistical software was used for analysis. Continuous data were presented as mean ± SD. Categorical data were presented as *n* (%). The Kappa statistic was used to evaluate the classification agreement between the MRI and intraoperative findings, and the inter-observer reliability between the two experienced observers for the MRI findings (based on a random sample of 55 cases). Interpretation of kappa values were considered poor (<0.00), slight (0.00–0.20), fair (0.21–0.40), moderate (0.41–0.60), substantial (0.61–0.80), or almost perfect (0.81–1.00) agreement. Additionally, the sensitivity, specificity, accuracy, and their respective 95% confidence intervals were calculated for each tear location. Statistical significance was defined as *P* < 0.05.

## 3 Results

A total of 226 patients (84 female, 37.2%; mean age, 38.3 ± 12.8 years) were included in the study. Mean time from injury to MRI in this patient cohort was 6.5 ± 2.7 days. A detailed description of demographic parameters can be found in Table 3.

**Table 3:** Demographic patient characteristics.

Characteristic	Data (*n* = 226)
Age (years), y	38.3 ± 12.8
Sex, female, *n* (%)	84 (37.2%)
Body mass index, kg/m^2^	26.0 ± 4.5
Time from injury to MRI, d	6.5 ± 2.7

The agreement across MRI results between the two observers was good (*k* = 0.63, *P* < 0.001). There was substantial agreement between MRI-based classification and the gold standard across the 226 cases (*k* = 0.92, *P* < 0.001; Table 4). Specifically, the validity of the classification was supported by substantial to almost perfect agreement (k = 0.79–0.97) in well-sampled subtypes (*n* ≥ 15). For subtype 1a (*n* = 10), kappa was 1.00 with a 95% confidence interval of (1.00–1.00), which should be interpreted cautiously due to the limited sample size. Subtype 2b (*n* = 1) was not analyzed for agreement because of insufficient cases, and subtype 2a had no cases in either classification. MRI diagnostic performance for each of the seven locations is detailed in Table 5. Sensitivity ranged from 81.8% (95% CI: 47.8%–96.8%) for location S2 to 100% for locations P1 (95% CI: 97.0%–100%) and P2 (95% CI: 75.9%–100%). Specificity was consistently high, ranging from 90.8% (95% CI: 80.3%–96.2%) for D1 to 100% (95% CI: 97.8%–100%) for S2. Accuracy ranged from 95.6% (95% CI: 92.1%–97.6%) for D1 to 99.6% (95% CI: 97.5%–99.9%) for P1 and P2.

**Table 4:** Agreement between MRI and intraoperative classification of MCL tears.

MRI classification (*n*)	Intraoperative classification (*n*)							Total (*n*)	Kappa (95%CI)	*P*
	1a	1b	2a	2b	3	4a	4b			
1a	10	–	–	–	–	–	–	10	1.00 (1.00–1.00)^a^	0.000
1b	–	141	–	–	–	–	2	143	0.97 (0.94–1.00)	0.000
2a	–	–	–	–	–	–	–	–	–^b^	–
2b	–	–	–	1	–	–	–	1	–^c^	–
3	–	–	–	–	27	–	–	27	0.87 (0.77–0.96)	0.000
4a	–	–	–	–	5	10	–	15	0.79 (0.61–0.97)	0.000
4b	–	1	–	–	2	–	27	30	0.90 (0.82–0.99)	0.000

a Interpret cautiously due to small sample size (*n* = 10).

b No cases available for subtype 2a.

c Insufficient sample (*n* = 1) to reliably estimate kappa.

**Table 5:** Accuracy of MRI in diagnosing tear locations against the surgical gold standard.

Tear location	TN	FP	FN	TP	Sensitivity (%, 95% CI)	Specificity (%, 95% CI)	Accuracy (%, 95% CI)
S1	71	3	5	147	96.7% (92.1%–98.8%)	95.9% (87.8%–98.9%)	96.5% (93.2%–98.2%)
S2	215	0	2	9	81.8% (47.8%–96.8%)	100.0% (97.8%–100.0%)	99.1% (96.8%–99.8%)
S3	164	2	1	59	98.3% (89.9%–99.9%)	98.8% (95.3%–99.8%)	98.7% (96.2%–99.6%)
D1	59	6	4	157	97.5% (93.4%–99.2%)	90.8% (80.3%–96.2%)	95.6% (92.1%–97.6%)
D2	208	3	1	14	93.3% (66.0%–99.7%)	98.6% (95.6%–99.6%)	98.2% (95.5%–99.3%)
P1	67	1	0	158	100.0% (97.0%–100.0%)	98.5% (91.0%–99.9%)	99.6% (97.5%–99.9%)
P2	209	1	0	16	100.0% (75.9%–100.0%)	99.5% (97.0%–100.0%)	99.6% (97.5%–99.9%)

TP, true positive; FP, false positive; FN, false negative; TN, true negative. Sensitivity = TP/(TP + FN), specificity = TN/(TN + FP), accuracy = (TP + TN)/total. 95% confidence intervals were calculated using the Wilson score method.

The distribution and frequency of MRI findings are presented in Table 6. Among the assessed tear types, type 1b was the most prevalent (63.3%, *n* = 143), followed by type 4b (13.3%, *n* = 30) and type 3 (11.9%, *n* = 27). In contrast, type 2b was the least common finding (0.4%, *n* = 1), and no type 2a injuries were present in this cohort. Given that the two most common injury patterns (types 1b and 4b) both involved the POL, we further analyzed the prevalence of tears in this critical structure. POL tears were present in 77.0% (*n* = 174) of the cohort.

**Table 6:** MRI findings in the study cohort (*n* = 226).

Tear Type	Patients, *n* (%)
1a	10 (4.4%)
1b	143 (63.3%)
2a	0 (0.0%)
2b	1 (0.4%)
3	27 (11.9%)
4a	15 (6.6%)
4b	30 (13.3%)

## 4 Discussion

This study introduces a classification system designed to evaluate tear locations in MCL tears using MRI. This classification not only possesses a solid foundation in anatomical and biomechanical theory (19, 26, 31), but also demonstrated excellent inter-observer reliability and diagnostic accuracy in MRI. Applying this classification, our study revealed that the identification of a combined POL tear or distal tibial-side MCL tear on MRI can serve as a key factor for MCL repair surgery. This finding confirms the crucial role of MRI-based localization in assessing MCL injury severity and guiding surgical decision-making.

The classification system is based on the established anatomy of the MCL, comprising the sMCL, dMCL, and POL (2, 23, 32). Using the seven ligamentous attachments as anatomical landmarks, we identified tear locations on MRI. Tears involving the adjacent femoral attachments of the sMCL and dMCL (S1 and D1) frequently occurred concomitantly (81% in this cohort), reflecting their close anatomical relationship (32). However, MRI diagnosis based specifically on these two attachments (S1 and D1) may be subject to minor inaccuracies. The sMCL has two tibial attachments (proximal and distal) with distinct biomechanical roles (19). On MRI, the proximal attachment (S2) is often difficult to distinguish from the adjacent dMCL attachment (D2). Isolated POL injury was rare, consistent with previous reports (28, 31), and no such case was observed in our surgical cohort. The femoral attachment of the POL is located posterior to that of the sMCL (32). Its primary distal attachments include the tibia, the posterior horn of the medial meniscus, the semimembranosus tendon sheath, and the posteromedial joint capsule (12, 23). Given the anatomical complexity and close proximity of these distal attachments, which are difficult to differentiate on MRI, we collectively refer to this region as the distal tibial side or semimembranosus side.

Based on these anatomical distributions, we grouped the seven attachments into three areas (femoral, proximal tibial, and distal tibial) corresponding to types 1, 2, and 3, respectively. Combined tears involving more than one area were classified as type 4. Tears involving the sMCL and/or dMCL were designated subtype a, and those involving the POL were designated subtype b. The inter-observer reliability between the two experienced observers was good (K = 0.63), indicating substantial agreement and supporting the reproducibility of the classification system.

Meanwhile, based on the aforementioned seven attachments, the study also collected intraoperative records from patients as the gold standard, and compared them with preoperative MRI findings. MRI demonstrated high diagnostic performance across all locations, with sensitivity ranging from 81.8% to 100%, specificity from 90.8% to 100%, and accuracy from 95.6% to 99.6%. Notably, the highest performance was observed for the P1 and P2 locations, while the lowest were for S2 and D1. The classification agreement is also excellent. This is in close agreement with the established literature, which has reported a diagnostic accuracy of 94% for detecting injuries of the knee collateral ligaments (24). In individual cases of inconsistency, it is mainly due to the difficulty in distinguishing S1 from D1 and S2 from D2 in MRI.

Particularly notable was the high diagnostic accuracy for POL. In our experience, the adductor tubercle is reliably identified by first locating the adductor magnus tendon on consecutive MRI slices. Following this, the anatomical regions are defined as follows: POL encompasses the adductor tubercle itself and all slices posterior to it, while sMCL includes the slices anterior to the adductor tubercle. As demonstrated by Sanghvi et al. and Chapman et al., the adductor magnus tendon, being rarely injured, serves as an important surgical landmark for identifying the femoral attachments of the sMCL and POL, with the POL originating near the adductor tubercle from the sMCL's posterior margin (7, 33).

In our study, MRI revealed that Grade III MCL injuries were most frequently associated with femoral-side tears involving the POL and the sMCL and/or dMCL (Type 1b), followed by multi-region tears involving the POL and the sMCL and/or dMCL (Type 4b). These findings show that most Grade III MCL injuries are often associated with POL, particularly on the femoral side. Previous studies have also mentioned severe valgus instability in Grade III injuries, which is caused by tears in the sMCL and dMCL, especially the rupture of the POL (7, 10, 34, 35). This is related to the function of the POL, which protects the knee joint against valgus stress in the range of 0°–30° and internal rotation, so that instability at both 0° and 30° is more likely to occur after POL torn (35, 36).

Sims and Jacobson found that 99% of patients with medial knee surgery had an injury of the POL (12). Some authors have suggested that the POL should be considered part of the posteromedial corner of the knee (1, 37, 38). Differences in anatomical understanding can lead to different descriptions in diagnosis and treatment guidelines. The statement in the literature that isolated grade III MCL injuries do not require operation is likely based on the understanding that MCL does not include POL, and its diagnosis is mostly graded according to 30° valgus stress (3, 18, 24). However, there is limited literature on POL and radiological findings of POL injury. Therefore, despite its importance, it is rarely considered in clinical diagnosis, especially in complex knee injuries (33, 38).

Among the 23% of cases without concomitant POL tear, distal tibial-side tears accounted for 51.9%. Therefore,distal tibial-side tears were also commonly observed in Grade III MCL injuries, which corresponds to the indications for MCL surgery described in numerous literature reports (3, 23, 38, 39).

MRI is reasonably accurate in determining the location of injuries (2, 19, 24). It can indicate whether the MCL is ruptured or not, and it can also show the exact location of the injury as well as concomitant other ligament, soft tissue, or bone injuries (11, 24, 25), especially in patients with acute MCL injuries (23). MRI serves as a supplementary method for diagnosing grade III injuries (2, 5, 11, 23). In most literature,the MRI classification defines three grades: grade I indicates an intact ligament with the presence of periligamentous edema, grade II indicates a partial tear with surrounding edema, and grade III indicates a complete tear of the ligament (19). The above classification determines the severity of tear based on signal intensity, but does not specify the location of the MCL tear. Importantly, it is unable to quantify the degree of pathologic laxity due to ligament rupture (31). This study instead employed MRI to diagnose tear locations, investigating the relationship between the Grade III injuries and corresponding MRI findings.

Hadi Makhmalbaf et al. (2) classified MCL injury locations based on MRI findings, describing the MRI characteristics and treatment strategies for each type. This study partially addressed diagnostic and therapeutic challenges for MCL injuries. However, it lacked supporting data and did not comprehensively cover all MCL injury types. To conclude, most literature discussing non-surgical and surgical treatments for MCL injuries fails to differentiate the location of ligament injury and lacks intraoperative validation for MRI-based descriptions of MCL injury (3, 11). The present study partially alleviates these prior limitations.

This study has several limitations. First, the cohort included only surgically treated patients, introducing selection bias by excluding conservatively managed cases. Second, diagnostic accuracy was based on consensus interpretations by two observers. While this minimized subjective bias, it may overestimate the performance of individual radiologists in routine practice. Future studies should include multiple independent readers with varying experience levels. Finally, MRI data were acquired using scanners with different field strengths and manufacturers. Although this variability is a potential limitation, previous studies suggest that it does not significantly affect diagnostic accuracy for ligament tears (29, 40). Prospective multicenter studies with standardized imaging protocols and clinical follow-up are needed to validate our findings.

Just as some scholars compare the POL to a sail, we find that the structure of the MCL closely resembles the topography of South America, with the dMCL and sMCL corresponding to the Mount Andes and the POL to the Brazilian Highlands.The longitudinal fibers of the superficial medial collateral ligament resemble the majestic Andes, towering and robust, whereas the posterior oblique fibers spread out like the expansive Brazilian Highlands, broad yet resilient in maintaining posteromedial knee stability.

## 5 Conclusion

The presence of a POL tear is a key factor in determining whether MCL repair surgery is necessary. MRI has demonstrated to be highly effective in locating MCL tears, particularly those involving the POL. This study highlights the value of MRI in assessing MCL injury severity and guiding surgical decision-making, with the adductor tubercle serving as a reliable reference for identifying POL tears. Prospective studies with larger cohorts are warranted to further validate these findings and determine how this classification system influences surgical decision-making.

## Data Availability

The raw data supporting the conclusions of this article will be made available by the authors, without undue reservation.

## References

[1] Madi S Acharya K Pandey V. Current concepts on management of medial and posteromedial knee injuries. J Clin Orthop Trauma. (2022) 27:101807. 10.1016/j.jcot.2022.101807 35242534 PMC8873958

[2] Makhmalbaf H Shahpari O. Medial collateral ligament injury; a new classification based on MRI and clinical findings. A guide for patient selection and early surgical intervention. Arch Bone Joint Surg. (2018) 6(1):3–7. 29430488 PMC5799597

[3] Trofa DP Sonnenfeld JJ Song DJ Lynch TS. Distal knee medial collateral ligament repair with suture augmentation. Arthrosc Tech. (2018) 7(9):e921–6. 10.1016/j.eats.2018.05.001 30258773 PMC6153306

[4] Vosoughi F Rezaei Dogahe R Nuri A Ayati Firoozabadi M Mortazavi J. Medial collateral ligament injury of the knee: a review on current concept and management. Arch Bone Joint Surg. (2021) 9(3):255–62. 10.22038/abjs.2021.48458.2401 34239952 PMC8221433

[5] Andrews K Lu A Mckean L Ebraheim N. Review: medial collateral ligament injuries. J Orthop. (2017) 14(4):550–4. 10.1016/j.jor.2017.07.017 28878515 PMC5581380

[6] Edson CJ. Conservative and postoperative rehabilitation of isolated and combined injuries of the medial collateral ligament. Sports Med Arthrosc. (2006) 14(2):105–10. 10.1097/01.jsa.0000212308.32076.f2 17135955

[7] Chapman G Vij N LaPrade R Amin N. Medial-sided ligamentous injuries of the athlete’s knee: evaluation and management. Cureus. (2023) 15(3):e36360. 10.7759/cureus.36360 37082476 PMC10112817

[8] DeLong JM Waterman BR. Surgical techniques for the reconstruction of medial collateral ligament and posteromedial corner injuries of the knee: a systematic review. Arthroscopy. (2015) 31(11):2258–2272.e1. 10.1016/j.arthro.2015.05.011 26194939

[9] Kannus P. Long-term results of conservatively treated medial collateral ligament injuries of the knee joint. Clin Orthop Relat Res. (1988) 226:103–12. 3335084

[10] Wijdicks CA Griffith CJ Johansen S Engebretsen L LaPrade RF. Injuries to the medial collateral ligament and associated medial structures of the knee. J Bone Joint Surg. (2010) 92(5):1266–80. 10.2106/JBJS.I.01229 20439679

[11] Meyer P Reiter A Akoto R Steadman J Pagenstert G Frosch KH Imaging of the medial collateral ligament of the knee: a systematic review. Arch Orthop Trauma Surg. (2022) 142(12):3721–36. 10.1007/s00402-021-04200-8 34628563 PMC9596543

[12] Sims WF Jacobson KE. The posteromedial corner of the knee. Am J Sports Med. (2004) 32(2):337–45. 10.1177/0363546503261738 14977657

[13] Robins AJ Newman AP Burks RT. Postoperative return of motion in anterior cruciate ligament and medial collateral ligament injuires. Am J Sports Med. (1993) 21(1):20–5. 10.1177/036354659302100104 8427364

[14] Fetto J Marshall J. Medial collateral ligament injuries of the knee: a rationale for treatment. Clin Orthop Relat Res. (1978) 132:206–18. 679543

[15] Kim C Chasse PM Taylor DC. Return to play after medial collateral ligament injury. Clin Sports Med. (2016) 35(4):679–96. 10.1016/j.csm.2016.05.011 27543407

[16] Noyes FR Barber-Westin SD. The treatment of acute combined ruptures of the anterior cruciate and medial ligaments of the knee. Am J Sports Med. (1995) 23(4):380–9. 10.1177/036354659502300402 7573644

[17] Blaber OK DeFoor MT Aman ZA McDermott ER DePhillipo NN Dickens JF Lack of consensus on the management of medial collateral ligament tears in the setting of concomitant anterior cruciate ligament injury. JBJS Rev. (2024) 12(6):1–11. 10.2106/JBJS.RVW.24.00036 38913807

[18] Petermann J von Garrel T Gotzen L. Non-operative treatment of acute medial collateral ligament lesions of the knee joint. Knee Surg Sports Traumatol Arthrosc. (1993) 1(2):93–6. 10.1007/BF01565459 8536015

[19] Encinas-Ullán CA Rodríguez-Merchán EC. Isolated medial collateral ligament tears. EFORT Open Rev. (2018) 3(7):398–407. 10.1302/2058-5241.3.170035 30233815 PMC6129956

[20] Phisitkul P. MCL injuries of the knee: current concepts review. Iowa Orthop J. (2006) 26:77–90. 16789454 PMC1888587

[21] Laprade RF Bernhardson AS Griffith CJ Macalena JA Wijdicks CA. Correlation of valgus stress radiographs with medial knee ligament injuries. Am J Sports Med. (2010) 38(2):330–8. 10.1177/0363546509349347 19966093

[22] Hastings DE. The non-operative management of collateral ligament injuries of the knee joint. Clin Orthop Relat Res. (1980) 147:22–8. 10.1097/00003086-198003000-00005 7371301

[23] Marchant MJ Tibor LM Sekiya JK Hardaker WJ Garrett WJ Taylor DC. Management of medial-sided knee injuries, part 1. Am J Sports Med. (2011) 39(5):1102–13. 10.1177/0363546510385999 21148144

[24] House CV Connell DA Saifuddin A. Posteromedial corner injuries of the knee. Clin Radiol. (2007) 62(6):539–46. 10.1016/j.crad.2006.11.024 17467390

[25] Nakamura N Horibe S Toritsuka Y Mitsuoka T Yoshikawa H Shino K. Acute grade III medial collateral ligament injury of the knee associated with anterior cruciate ligament tear. The usefulness of magnetic resonance imaging in determining a treatment regimen. Am J Sports Med. (2003) 31(2):261–7. 10.1177/03635465030310021801 12642263

[26] Von Rehlingen-Prinz F Rilk S Krishnan KR Tomanek F Beckers V Goodhart GC Tear location of superficial medial collateral ligament tears: validation of a magnetic resonance imaging–based classification system. Am J Sports Med. (2025) 53(6):1400–8. 10.1177/03635465251330005 40211692

[27] von Rehlingen-Prinz F Krishnan KR Rilk S Tomanek F Goodhart GC Beckers V Location of medial collateral ligament tears: introduction to a magnetic resonance imaging-based classification. Skeletal Radiol. (2025) 54(4):851–60. 10.1007/s00256-024-04747-8 39083055

[28] D’Ambrosi R Corona K Guerra G Cerciello S Ursino C Ursino N Posterior oblique ligament of the knee: state of the art. EFORT Open Rev. (2021) 6(5):364–71. 10.1302/2058-5241.6.200127 34150330 PMC8183151

[29] Nouri N Bouaziz MC Riahi H Mechri M Kherfani A Ouertatani M Traumatic meniscus and cruciate ligament tears in young patients: a comparison of 3T versus 1.5T MRI. J Belg Soc Radiol. (2017) 101(1):14. 10.5334/jbr-btr.1158 30039006 PMC5854230

[30] Ghiretti R Panzavolta F Lucidi GA Zaffagnini S. Combined ACL–MCL injuries: anatomy, biomechanics, and clinical management. Medicina (B Aires). (2025) 61(10):1788. 10.3390/medicina61101788 PMC12566525 41155775

[31] Cristiani R Van De Bunt F Kvist J Stålman A. High prevalence of superficial and deep medial collateral ligament injuries on magnetic resonance imaging in patients with anterior cruciate ligament tears. Arthroscopy. (2024) 40(1):103–10. 10.1016/j.arthro.2023.05.029 37353094

[32] LaPrade RF Engebretsen AH Ly TV Johansen S Wentorf FA Engebretsen L. The anatomy of the medial part of the knee. J Bone Joint Surg Am. (2007) 89(9):2000–10. 10.2106/JBJS.F.01176 17768198

[33] Sanghvi D Srivastav A Agrawal S Nakshiwala V. The posterior oblique ligament in MRI of acute knee trauma. Skeletal Radiol. (2022) 51(5):1063–71. 10.1007/s00256-021-03930-5 34626207

[34] Hughston J. The importance of the posterior oblique ligament in repairs of acute tears of the medial ligaments in knees with and without an associated rupture of the anterior cruciate ligament. Results of long-term follow-up. J Bone Joint Surg. (1994) 76(9):1328–44. 10.2106/00004623-199409000-00008 8077263

[35] Heitmann M Preiss A Giannakos A Frosch KH. Akute mediale seitenbandverletzung am kniegelenk. Unfallchirurg. (2013) 116(6):497–503. 10.1007/s00113-013-2371-8 23694961

[36] Lucidi GA Solaro L Grassi A Alhalalmeh MI Ratti S Manzoli L Current trends in the medial side of the knee: not only medial collateral ligament (MCL). J Orthop Traumatol. (2024) 25:69. 10.1186/s10195-024-00808-9 39704918 PMC11662134

[37] Lundquist RB Matcuk GR Jr Schein AJ Skalski MR White EA Forrester DM Posteromedial corner of the knee: the neglected corner. Radiographics. (2015) 35(4):1123–37. 10.1148/rg.2015140166 26172356

[38] Jungmann PM. Posteromediale ecke des kniegelenks. Radiologe. (2019) 59(2):155–68. 10.1007/s00117-018-0488-z 30645662

[39] Bollier M Smith PA. Anterior cruciate ligament and medial collateral ligament injuries. J Knee Surg. (2014) 27(5):359–68. 10.1055/s-0034-1381961 24949985

[40] Hergár L Kovács N Agócs G Weninger V Skaliczki G Lutz E No evidence for the superiority of 3 tesla magnetic resonance imaging over 1.5 tesla magnetic resonance imaging for diagnosing wrist ligamentous lesions: a systematic review and meta-analysis. Arthroscopy. (2024) 40(11):2730–2741.e10. 10.1016/j.arthro.2024.04.029 38735416

